# Socioeconomic predictors of preventive health check-ups by a general practitioner in the Czech Republic

**DOI:** 10.3389/fmed.2024.1341621

**Published:** 2024-12-23

**Authors:** Jarmila Zimmermannova, Jiri Vevoda, Tereza Schovankova, Ondrej Holy

**Affiliations:** ^1^Science and Research Centre, Faculty of Health Sciences, Palacký University Olomouc, Olomouc, Czechia; ^2^Department of Humanities and Social Sciences, Faculty of Health Sciences, Palacký University Olomouc, Olomouc, Czechia

**Keywords:** aging, preventive examinations, socioeconomic indicators, seniors, Czech Republic

## Abstract

Health is one of the Sustainable Development Goals. The importance of health promotion is growing in the context of an aging population and increasing life expectancy. Prevention is often underestimated and neglected by citizens. This article aims to identify the socioeconomic predictors of preventive health check-ups by general practitioners in the Czech Republic, focusing on selected age groups. An original dataset is prepared based on data for 2010–2019 provided by the largest health insurance company in the Czech Republic, the General Health Insurance Company. Correlation and regression analysis methods are used to achieve the objectives. Two models are built and tested: (1) preventive examinations model and (2) preventive examinations in age group 65+ model. Based on the results, preventive medical examinations in the pensioner group depended on economic indicators, such as the average wage, employment, and gross domestic product, in the analyzed period. For the total population, overall population size, the average age, urbanized area, and level of education play a key role. The results reveal a difference between the general population group and the 65+ population group. Government interventions and health policies promoting prevention should consider using appropriate incentive policy instruments targeting the 65+ population to prolong active life in senior age.

## 1 Introduction

Good health and wellbeing are one of the Sustainable Development Goals (1). As the focus of this goal implies, ensuring a healthy life and a healthy lifestyle are essential across all generations (2, 3).

Concerning health services accessibility, the Czech Republic has had a government-regulated social health insurance (SHI) system since the early 1990s. There are currently seven large insurance funds on the market (4), the largest of which is the General Health Insurance Company (5).

The General Health Insurance Company has been the leader of the Czech public health insurance system for more than 30 years and covers 56% of the population. It was established on 1 January 1992. Over time, other health insurance companies were established. However, with almost 6 million clients, it is still the largest health insurance company in the Czech Republic (6), even in competition with six others (7). Health insurance is compulsory, and healthcare access is available to all residents. The general package of services is the same for all inhabitants. Health insurance companies may offer different benefits, such as co-payments for non-mandatory vaccinations (8).

The availability of services varies widely between regions due to the capacity of doctors and nurses. In recent years, the Ministry of Health and health insurance funds have introduced targeted programmes to provide financial incentives for dentists, general practitioners and pharmacists to work in underserved areas. The strategic goal of improving the health of the population and reducing health inequalities is part of official ministerial documents (9). The current distribution of GPs supply is relatively even in the Czech Republic (10).

Based on Country Health Profiles 2023 (11), in the Czech Republic, poor diet, smoking and drinking alcohol are among the most significant health risks. In 2019, they accounted for almost half of all deaths. Obesity rates have been steadily increasing, hovering around 20% in adults. It contributes to the high prevalence of diabetes and other related diseases. Regarding alcohol, the Czech Republic is one of the EU countries with the highest per capita consumption (11).

The importance of health promotion is increasing in the context of behavioral risk factors, population aging, and longer active life expectancy (12). Within the public health insurance system of the Czech Republic (4), preventive health check-ups by general practitioners embody a fundamental tool for maintaining and enhancing public health. These check-ups aim to identify potential health issues, allowing for prompt medical intervention and prevention of more severe complications (13). Insured individuals are entitled to a preventive check-up once every two years, starting from the age of 18 or at the latest upon reaching 19 years of age (9).

The content of these health check-ups encompasses the establishment and update of a patient's medical history, assessment of familial medical history, prior illnesses, work strain, habits, and anticipated health risks (14). These health check-ups also include laboratory tests, such as basic chemical urine analysis and blood cholesterol and lipid tests at the ages of 18, 30, 40, 50, and 60. Furthermore, they incorporate ECGs at 40 years of age and 4-year intervals, fecal occult blood tests from 50 years of age, mammographic examinations for women from 45 years of age, and serum creatinine and glomerular filtration rate examinations for patients suffering from diabetes, hypertension, or cardiovascular complications from 50 years of age at 4-year intervals. Patients aged 45–61 are also provided with recommendations for preventive eye examinations at 4-year intervals (15).

Besides the general preventive health check-ups, gynecological preventive check-ups for women from 15 years of age once a year and dental preventive check-ups also once a year are conducted in the Czech Republic (15). All preventive health check-ups are fully covered by public health insurance (16).

Prevention is often underestimated and neglected by citizens (17). Preventive health check-ups focus predominantly on the early detection of chronic diseases, primary prevention, and health counseling (14). To secure health gains and prevent health inequalities, support for high and equitable uptake of such universal screening is essential (18).

Various case and expert studies have addressed the issue of prevention and the factors that influence the willingness of the population to undergo preventive health check-ups. Such studies have predominantly focused on socio-demographic, psychosocial, and medical indicators; however, only a few have had data on economic and education indicators. For the purpose of this article, the authors were particularly inspired by scientific papers dealing with socioeconomic and demographic factors.

Researchers in Germany (19) analyzed the social, behavioral, and health-related factors that impact the participation of eligible adults in health screenings. The study reviewed data from a national sample of 26,555 adults supplied by the cross-sectional German Health Update (GEDA) survey. The team utilized logistic regression models to determine the association between various factors and attendance at health check-ups.

Another study from Germany (20) examined whether healthcare inequality is responsible for differences in the utilization of prevention and health promotion services among various social classes, as determined by education, occupation, and income. Additionally, it investigated how health promotion and prevention efforts and research could be enhanced to reduce such disparities. To this end, the researchers conducted a systematic literature review to identify relevant articles.

An interesting study focuses on preventive health check-ups in Austria (14). The research investigates the predictors of adherence to the recommended interval of preventive health check-up performance, based on data from the Austrian Health Interview Survey 2006/07 (15,474 participants). The aim is to identify factors that impact compliance with recommended preventive health check-ups. The study employed participation in preventive health check-ups in the previous 3 years as the dependent variable and socio-demographic and health-related characteristics as independent variables. Regarding socio-demographic variables, participants who were middle-aged, had secondary education (women) or tertiary education (men), higher income, and were born in Austria (men) or another member state of the EU-15 (women) were more likely to have undergone a preventive health check.

Jørgensen et al. (21) analyzed the factors that determine frequent visits to general practitioners by the Danish adult population. They examined a range of lifestyle, socio-demographic, medical, and gender-specific factors. The study used data on visits to general practitioners from the Danish National Health Service Register at cohort baseline (1993–1997). The dataset comprised information on 54,849 individuals from the Danish Diet, Cancer and Health cohort (aged between 50 and 65 years), specifically regarding their visits to general practitioners. A questionnaire was used to gather information on medical conditions, lifestyle, socio-demographic, and gender-related factors. Regarding the influence of socio-demographic factors on frequent attendance, occupational status was found to be a significant determinant. Employed participants were 39% less likely to be frequent attendees compared to those who were unemployed. Additionally, an increase in educational level was negatively associated with the odds of being a frequent attendee.

Vončina et al. (22) examined the correlation between unemployment and the utilization of preventive healthcare services in Croatia. The researchers analyzed data on preventive healthcare services and employment status from the Croatia Adult Health Survey. The sample comprised 9,070 individuals from the general adult population in Croatia, which was representative of the larger population. The authors found that the unemployed received fewer preventive screenings and health services than the respective subgroups of the employed, whether or not they had cardiovascular and metabolic disease. Regarding non-EU countries, a research project undertaken in Saudi Arabia has emphasized the importance of preventive health screenings as a crucial strategy in combating numerous diseases. The study employed a national survey with an extensive sample of 11,528 participants, as specified by the authors. Nevertheless, several socioeconomic circumstances may impede extensive participation in this vital healthcare activity. The study findings indicate that there is a lack of documentation regarding the influence of socioeconomic disparities on this behavior (23).

Researchers in Korea (24) conducted a study to investigate sociodemographic and health-related factors that could influence health check-up participation among stroke survivors residing in the community. The authors worked with the Korea National Health and Nutrition Examination Survey and selected 642 stroke survivors as respondents. The constructed model considered sociodemographic factors, medical history, and health-related quality of life. In terms of sociodemographic factors, non-participants in health check-ups had significantly higher percentages of lower education, living alone and being unemployed than participants in health check-ups. Dryden et al. (18) conducted a literature review to examine general and preventive health checks as crucial components of modern anticipatory care policies. This review examined the sociodemographic, social-cognitive and clinical features of the population, with an emphasis on routine health assessments or preventive health assessments for cardiovascular disease.

The literature review shows evidence to support the relationship between socioeconomic factors and the use of preventive and health promotion services. On the other hand, there is a lack of studies focusing on specific factors influencing the senior population to undergo preventive screenings. As far as the Czech Republic is concerned, this issue has not yet been sufficiently investigated. No research has been conducted on the factors influencing the frequency of preventive examinations, either in the general population or in the population of seniors.

In the context of access to health care and equity in access to health care (2, 3), it is important to monitor the behavior of different population groups. In the Czech Republic, a fundamental feature of demographic development will be the significant aging of the population. This is shown by the new population projection for the Czech Republic covering the period 2023–2100. The proportion of the elderly will increase significantly. In the context of behavioral risk factors and increasing active life expectancy, the importance of health promotion in this group is growing.

A more detailed analysis of the 65+ population group is important for several reasons, both in terms of extending working life and increasing retirement age, as well as extending life expectancy both overall and in health and focusing on healthy lifestyles, active living, and wellbeing. Health check-ups can contribute to prolonging active life in the 65+ generation.

Therefore, the authors of this article try to identify factors influencing preventive check-ups in the Czech Republic, both in the general population and the population 65+, focusing on socioeconomic and demographic variables.

## 2 Materials and methods

### 2.1 Data

The original dataset based on annual data for the period 2010–2019 provided by the largest health insurance company in the Czech Republic (General Health Insurance Company) is used. Data is also available for 2020, but here it is affected by COVID-19 and therefore not included in the analysis. The data were aggregated from regions to the whole Czech Republic and further by age groups and include the number of preventive examinations by a general practitioner (GP).

The total number of preventive check-ups with a general practitioner had an increasing trend during the period under review, ranging from 822,097 in 2010 to 1,005,958 in 2019 for the total population (0–85+ years). In the 65+ population, the total number of preventive examinations by a general practitioner fluctuated over the study period, ranging from 223,174 in 2010 to 344,174 in 2019.

The key variables (preventive check-ups in the whole population and preventive check-ups in the group 65+) were provided by the insurance company in a structure according to age and by individual regions of the Czech Republic. Subsequently, for the purposes of the presented research, they were aggregated to the whole Czech Republic and into groups by age.

Besides data provided by General Health Insurance Company, other data describing various demographic and socioeconomic indicators in the Czech Republic were used from Eurostat (25) and the Czech Statistical Office (26).

Table 1 provides an overview of all relevant variables, their abbreviations, units, roles, and their assumed individual (pairwise) effects on frequency of preventive check-ups. Table 2 presents descriptive statistics of all relevant variables.

**Table 1:** List of variables.

**Variable**	**Abbreviation**	**Units**	**Role**	**Expected/theoretical effect**
Preventive medical check-ups (GP)	PRE	Persons	Dependent	–
Preventive medical check-ups in the group 65+ (GP)	PRE65	Persons 65+	Dependent	–
Share of urban population	URB	%	Independent	Positive
Population, total (as at 31 December)	POP	Persons	Independent	Positive
Average age (as at 31 December)	AGE	Years	Independent	Positive
GDP per capita	GDP	CZK, current prices	Independent	Positive
Basic education	BED	Persons	Independent	Negative
Secondary education	SED	Persons	Independent	Negative
Secondary education with graduation	SGED	Persons	Independent	Positive
Higher education	HED	Persons	Independent	Positive
Unemployment rate	UNE	%	Independent	Not clear
Average gross monthly wage per employee	AWA	CZK, current prices	Independent	Positive
Time	TIME	Years	Control	Positive

Source: authors' own elaboration.

**Table 2:** Descriptive statistics.

	***MIN***	***MAX***	***MEDIAN***
PRE	822,097	1,005,958	944,587.5
PRE65	223,174	344,174	252,438
URB	68.92	69.96	69.29
POP	10,505,445	10,693,939	10,546,059
AGE	40.80	42.48	41.78
GDP	358,956.77	538,816.24	418,424.6
BED	1,206,511	1,487,653	1,259,943
SED	2,913,155	3,191,021	3,054,184
SGED	3,004,943	3,086,562	3,044,151
HED	1,236,301	1,730,853	1,564,536
UNE	2.02	7.28	5.58
AWA	22,663	31,032	24,566
TIME	2,010	2,019	2,014.5

Source: authors' own elaboration.

The particular variables and the foundation for their employment in the models introduced are described in the next chapter.

### 2.2 Methodology

The main goal of this research is the identification of socioeconomic predictors of preventive health check-ups by a general practitioner (GP) in the Czech Republic, with a focus on selected age groups. In the context of this objective, the following research questions were established:

(RQ1): Do socioeconomic factors influence preventive check-ups by a general practitioner (GP) in the Czech Republic?
(RQ2): Are there differences in socioeconomic factors influencing preventive check-ups by a general practitioner (GP) by age group?

The research presented here uses a macroeconomic perspective. In order to achieve the stated research objectives, we used the methods of literature search, time series analysis, correlation analysis as well as regression analysis.

First, we performed a simple analysis of the collected data and a time series analysis. The data were also evaluated in terms of the characteristics of each variable. Subsequently, correlation analysis was performed using the Pearson correlation coefficient (PCC; see Table 3), supplemented by analysis using Spearman's coefficient (SRCC; see Table 4) (27). For correlation analysis, all variables contained in Tables 1, 2 were used. Then, we performed a more complex regression analysis. In the regression analysis, individual regression models were created so that we could observe the partial relationships of the selected variables. Thus, regression analysis, unlike bivariate correlation analysis, allows us to observe relationships between the dependent variable and multiple independent variables.

**Table 3:** Correlation analysis—the whole population.

	***PCC***	***SRCC***
URB	−0.976^*^	−1.000
POP	0.800^*^	0.915^*^
AGE	0.989^*^	1.000
GDP	0.915^*^	0.988^*^
BED	−0.983^*^	−0.952^*^
SED	−0.954^*^	−0.988^*^
SGED	−0.321	−0.079
HED	0.993^*^	0.988^*^
UNE	−0.905^*^	−0.964^*^
AWA	0.837^*^	0.988^*^
TIME	0.974^*^	1.000

^*^ p-value < 0.05.

Source: authors' own elaboration.

**Table 4:** Correlation analysis— population 65+.

	***PCC***	***SRCC***
URB	−0.514	−0.297
POP	0.493	0.091
AGE	0.465	0.297
GDP	0.505	0.285
BED	−0.382	−0.127
SED	−0.303	−0.200
SGED	−0.210	−0.430
HED	0.390	0.200
UNE	−0.351	−0.236
AWA	0.597	0.285
TIME	0.476	0.297

Source: authors' own elaboration.

Based on the research questions defined above, we created the following models:

1) Preventive examinations model (PRE model),
2) Preventive examinations in the age group 65+ model (PRE65 model). PRE model (1) or PRE65+ model (2) represents the relationship between preventive examinations in the whole population (Y-PRE) or preventive examinations in the age group 65+ (Y-PRE65) and selected variables. Both models are based on the period 2010–2019. The regression equation of these models can be described in detail as follows:

(1) $$\begin{array}{r} \text{PRE}/\text{PRE}65={\text{β}}_{0}+{\text{β}}_{1}\times \text{URB}+{\text{β}}_{2}\times \text{POP}+{\text{β}}_{3}\times \text{AGE}\\ +{\text{β}}_{4}\times \text{GDP}+{\text{β}}_{5}\times \text{BED}+{\text{β}}_{6}\times \text{HED}+{\text{β}}_{7}\times \text{UNE}\\ +{\text{β}}_{8}\times \text{AWA}+\text{u } \end{array}$$

where: Y-PRE [preventive examinations by a general practitioner (GP), in persons per year] or Y-PRE65+ [preventive examinations by a general practitioner (GP), in persons 65+ per year]; X_1_–URB (share of urban population); X_2_–POP (population in total); X_3_–AGE (average age); X_4_–GDP (GDP per capita); X_5_–BED (basic education); X_6_–HED (higher education); X_7_–UNE (unemployment rate), X_8_–AWA (average gross monthly wage), u—random element of the model.

The major reasons for the selection of relevant variables can be described as follows:

- URB: expected positive effect. Based on the analyses already carried out (28), a higher share of an urban population provides better accessibility to health care and health screening options for citizens.
- POP: expected positive effect. We assume that the increase in the total population includes a corresponding increase in the total number of health examinations.
- AGE: expected positive effect. Based on previous research (14), middle-aged and older citizens are more proactive about preventive check-ups.
- GDP: expected positive effect. Based on published studies (2, 3), it is expected that as the economic situation in countries improves, there will be greater support for the quality of healthcare, health check-ups as well as the well-being of the population.
- BED: expected negative effect. Previous research (14, 20, 24) has found that citizens with higher education are more likely to attend preventive check-ups than citizens with primary education.
- HED: expected positive effect. The explanation is similar to BED.
- UNE: non clear effect. Previous national studies have shown that unemployment is an important factor influencing health examinations, but the results are inconclusive as to the sign of this influence. While in Denmark and Korea (21, 24), the effect of unemployment on health examinations was found to be negative, in Croatia (22) was positive.
- AWA: expected positive effect. Based on scientific studies (14, 20), respondents with higher income were more likely to have undergone a preventive health check.

In addition to these variables, other factors may have a potential effect on PRE, such as the occupation of citizens, health policy changes, pandemics, disasters, and possibly others. As there are other factors having a potential effect on PRE, not included in the constructed models, it is essential to keep this in mind when interpreting the results.

Concerning the parameters of selected variables, diagnosis of multicollinearity was performed, based on the variance inflation factor (VIF). As the main models showed high values of VIF, it was decided to reduce the number of variables in the model and create sub-models. On the basis of the conducted literature review, three sub-models DEMO, ECO, and EDU were created for both studied population groups, i.e. for the general population and the 65+ population.

DEMO model consists of variables related to demography, such as URB, POP, and AGE. ECO model relies on variables related to economic characteristics, such as GDP, UNE, and AWA. EDU model includes variables characterizing the education level, such as BED and HED. All models contain tests for overall significance and autocorrelation. The *F*-test assessed the suitability of the regression models. The Durbin-Watson (DW) test was employed to examine the presence of autocorrelation.

## 3 Results

### 3.1 Correlation analysis

The correlation analysis was performed using available data and selected variables for the entire period 2010–2019. The correlation analysis was used to measure the relationship between the number of preventive examinations by a general practitioner (GP) in the whole population and the age group 65+ and other selected socio-economic variables. Table 3 presents the results of the correlation analysis (both PCC and SRCC).

From the results of the correlation analysis, we see a statistically significant positive correlation between the number of preventive examinations by a general practitioner (GP) in the whole population and POP, AGE, GDP, HED, AWA, and control variable TIME. Regarding the negative relationship, we can observe a statistically significant negative correlation with URB, BED, SED, and UNE. Based on these results, we can tentatively say that economic factors (higher GDP, higher average wages, lower unemployment) as well as education level and age have a positive effect on preventive examinations.

The results are similar for the correlation analysis performed using Pearson (PCC) and Spearman (SRCC) correlation coefficients.

If we focus on the population 65+, we obtain the results presented in Table 4.

As can be seen from the correlation analysis performed, the relationship between the number of examinations and the individual indicators is slightly different in pensioners than in the general population. The results are not statistically significant, but they show us at least a positive or negative relationship between the variables. In terms of the individual correlation coefficients, we see a higher weight of the economic indicators compared to the demographic ones. We will subsequently look at this in more detail in the regression analysis.

When interpreting the results of the correlation analysis, it is important to note that it is a bivariate relationship. Thus, we consider the effect of one variable on the other, without including the effect of the other variables. Thus, we can say that in the Czech Republic the proportion of urban population has a rather negative effect on preventive examinations, while a growing population, higher age, higher average wage and economic growth have a positive effect. With regard to the education of the population, higher education has a positive effect, while primary and secondary education have a negative effect. Also the increase in unemployment has a negative effect on the number of preventive examinations. Here we are talking about the total population, for the 65+ population the correlations between the variables are not statistically significant.

When considering more complex, simultaneous effects of the chosen variables, more complex regression models need to be developed and tested. These models will be presented in the following subsections.

### 3.2 Preventive examinations model

Firstly, we will try to answer RQ1 and observe the statistically significant influence of selected socioeconomic and demographic factors on preventive health check-ups by a general practitioner (GP) in the Czech Republic. We will work with the PRE model and its sub-models.

Concerning the results of correlation analysis, more sophisticated regression analysis serves to identify relationships between the variables according to their characteristics, such as demographic, economic, and educational variables. The individual sub-models are based on groups of selected variables. Thus, we will work with a model containing demographic indicators (DEMO), economic indicators (ECO), and educational indicators (EDU). Table 5 provides an overview of results for PRE model and its sub-models.

**Table 5:** Regression analysis—PRE model.

	**PRE MODEL**		**PRE-DEMO**		**PRE-EKO**		**PRE-EDU**		**PRE-EDU2**	
	**Sig**.	**Coef**.	**Sig**.	**Coef**.	**Sig**.	**Coef**.	**Sig**.	**Coef**.	**Sig**.	**Coef**.
URB	0.51	−774,287	0.051	119,474.12	–		–		–	
POP	0.48	2.93	0.006	−0.32	–		–		–	
AGE	0.79	75,704.44	0.001	213,930.82	–		–		–	
GDP	0.48	5.63	–		0.010	1.97	–		0.824	0.03
BED	0.46	4.03	–		–		0.473	−0.11	–	
HED	0.47	1.75	–		–		0.011	0.31	0.000	0.37
UNE	0.46	103,171.9	–		–		–		–	
AWA	0.46	−171.172	–		0.107	−24.51	–		–	
Const.	0.73	13,900,789	0.02	−12,952,185	0.00	715,087	0.11	602,459	0.00	354,952
Observ.	10		10		10		10		10	
*R* ^2^	0.99		0.99		0.94		0.99		0.99	
Signif.	0.10		0.00		0.00		0.00		0.00	
DW	–		3.24		0.97		2.22		2.085	

Source: authors' own elaboration,

If we look at the PRE model containing all the selected variables, it is clear that the model as a whole is not statistically significant with a significance level of 0.1. Moreover, the model is characterized by high multicollinearity (VIF). On the other hand, the simultaneous effect of all independent variables on the dependent variable PRE shows us the direction of this effect. It is interesting to observe the negative effect of the URB and AWA variables and the positive effect of all other variables in the model. Individual sub-models of the PRE model, such as PRE-DEMO, PRE-EKO, PRE-EDU, and PRE-EDU2, represent statistically significant models with *p* < 0.01 and an acceptable level of multicollinearity. The results suggest a coefficient of determination that is considerably high in the models. Variables with a *p*-value below 5% are significant, such as URB, POP, AGE, GDP, and HED. AGE, GDP and HED have a positive effect in all sub-models.

Based on the Durbin–Watson test (DW) results, no autocorrelation is visible for EDU and EDU2 sub-model. For other sub-models, it is also acceptable. The DW value is in between the critical values.

When interpreting the results of the regression analysis, it is important to note that it is more complex relationship, not just bivariate (as in correlation analysis), the individual variables in the models come out with different signs. This is due to the interactions of the variables.

Looking at the PRE model and all the statistically significant sub-models listed in Table 5, we can highlight that in the general population in the Czech Republic, increasing age, higher GDP and higher level of education have a positive effect on preventive examinations. This effect occurs both in the main PRE model and in its sub-models. Concerning other variables, their influence in each model varies according to the model and characteristics of the other variables.

### 3.3 Preventive examinations in the age group 65+ model

To answer the second research question, we first need to look at the behavior of the 65+ population and then compare the results with the whole population.

Similar to the PRE model we performed a regression analysis using the selected variables. The main model PRE65+ and its sub-models focus on individual characteristics of the population, such as demographic, economic, and educational indicators.

In the 65+ population, only the economic sub-model and economic variables such as GDP, AWA, and UNE have an impact. The results are shown in Table 6.

**Table 6:** Regression analysis—PRE 65+ model.

	**PRE65**+ **MODEL**		**PRE65**+ **DEMO**		**PRE65**+ **ECO**		**PRE65**+ **ECO2**		**PRE65**+ **EDU**	
	**Sig**.	**Coef**.	**Sig**.	**Coef**.	**Sig**.	**Coef**.	**Sig**.	**Coef**.	**Sig**.	**Coef**.
URB	0.99	−41,589.4	0.09	−454,280	–		–		–	
POP	0.96	−0.49	0.14	0.60	–		–		–	
AGE	0.66	451,852.3	0.10	−287,846	–		–		–	
GDP	0.96	0.87	–		0.12	1.568	–		–	
BED	0.98	0.243	–		–		–		0.98	0.01
HED	0.87	−11,106	–		–		–		0.31	0.08
UNE	0.93	34,555.17	–		0.02	52,709.72	0.03	26,647.57	–	
AWA	0.99	2.92	–		0.39	10.526	0.01	27.28	–	
Const.	0.94	−9,656,948	0.11		0.02	−949,694	0.04	−574,136	0.94	101,982.9
Obs.	10		10		10		10		10	
*R* ^2^	0.95		0.74		0.89		0.83		0.39	
Signif.	0.60		0.16		0.02		0.01		0.56	
DW	–		2.19		2.53		1.98		1.63	

Source: authors' own elaboration.

Individual sub-models of the PRE65 model, such as PRE-DEMO, PRE-EKO, PRE-EDU represent statistically significant models with *p* < 0.05.

Variables with a significance level of below 5% include GDP, UNE, and AWA. The DW test indicates no presence of autocorrelation.

In the 65+ economic model, the number of health check-ups is affected by UNE, GDP, and AWA.

When interpreting the results of the regression analysis, it is important to note that we focused primarily on selected variables. However, there can be other variables with the impact on behavior of population 65+, such as comorbidity, social support (the role of family, friends, and caregivers), technological barriers, fear, anxiety, and others.

Looking at all the models listed in Table 6, we can highligh that concerning economic factors, increasing GDP (economic growth), average wage and unemployment have a positive effect on preventive examinations. Concerning GDP and average wage, these indicators are associated with the overall economic level/activity and are also reflected in the 65+ generation.

In the case of unemployment, it can be understood that if unemployment increases in a society, there are also layoffs in the 65+ generation, and the elimination of part-time jobs, agreements and part-time jobs for the seniors. In this case, the increasing number of preventive check-ups can be understood to mean that seniors are going to the doctor more for preventive check-ups, as they see them as a kind of social interaction, communication and to help them against loneliness.

Comparing the results for the general population and the 65+ population, we see that for the elderly group, the economic variables are the most important, all of which have a positive effect on preventive examinations. The results compared to the general population also differ for the demographic and education indicators, which do not play such a key role for the seniors.

## 4 Discussion

### 4.1 Principal findings

First of all, it should be underlined that in the Czech Republic, as well as in other European countries, a fundamental feature of demographic development will be the significant aging of the population. The proportion of seniors will increase significantly. In the context of behavioral risk factors and increasing active life expectancy, the importance of health promotion in this group is growing. Therefore, a more detailed analysis of the behavior of the 65+ population is crucial for several reasons, such as the extension of working life and retirement age, the increase in life expectancy and active healthy life. Health check-ups can improve the quality of life of the 65+ generation and it is important to monitor the factors that may influence this.

We begin with a discussion in relation to the research questions.

(RQ1): Do socioeconomic factors influence preventive check-ups by a general practitioner (GP) in the Czech Republic?

If we focus on the total population in the Czech Republic, the results show that socioeconomic factors have a significant impact on the attendance at preventive check-ups. These include indicators such as GDP, average wage, and unemployment. However, in addition to socioeconomic factors, demographic and geographic factors such as population size, average age, and proportion of urban population are also significant in models focusing on the total population. Urban and rural disparities can influence access to healthcare resources. The level of education in society is also important, and the analysis shows a clear effect of higher levels of education on attendance at health check-ups.

(RQ2): Are there differences in socioeconomic factors influencing preventive check-ups by a general practitioner (GP) by age group?

The analysis carried out shows that there are differences between the selected age groups. The 65+ group is influenced by socioeconomic factors more than the rest of the population. If we look at the influence of other variables, even within the sub-models, it is clear that demographic characteristics and education level do not play a key role. The exception is the nature of urban and rural areas, which we focus on below.

The most important socioeconomic factors influencing the 65+ population's attendance at preventive check-ups are GDP, average wage, and unemployment. Within these variables, GDP and AWA have positive coefficients both in correlation analysis and regression analysis. This can be explained by the fact that a better economic situation and the higher average wage in society encourage the attendance of preventive check-ups among the retired group.

This can be related to the phenomenon of working pensioners. The popularity of working in retirement is increasing in the Czech Republic. Based on CZSO (30), the number of working pensioners in Czechia has more than doubled in 10 years (in the period 2011–2021). The number of working pensioners represented 10.7% of the total employed population in the year 2021. Concerning economic aspects, pensioners can receive a retirement pension and a salary at the same time. Working pensioners can therefore have wages from employment, business and capital income or rent at the same time. In any case, the amount of income from employment does not reduce the state pension while working.

What is also interesting for the 65+ population is that the URB coefficient is always negative in both the correlation analysis and the regression analysis (for the main model and its sub-models). This can be explained by the fact that in areas with high urban population density, pensioners attend fewer preventive check-ups than in rural areas.

### 4.2 Comparison with existing literature

The literature review suggests that there is evidence to support a relationship between socioeconomic factors and the use of preventive and health promotion services. On the other hand, the current scientific literature lacks studies focusing on specific factors influencing the senior population's uptake of preventive screenings. The comparison presented in this chapter therefore mostly refers to the results for the total population.

It should be emphasized that many studies work with data collected from various questionnaire surveys. For example, one of the few studies that also examines different population groups, including the group of seniors (14), works with data obtained from the Austrian Health Interview Survey database (AT-HIS) 2006–2007. The interviews were conducted face-to-face using computer assisted personal interviewing. In contrast, the present study for the Czech Republic is based on health examination data obtained from the largest health insurance company (General Health Insurance Company). The original dataset includes health check-ups completed between 2010 and 2019 (the year 2020 was omitted from the analysis due to COVID-19). Focusing on research dealing with group of pensioners, Brunner-Ziegler et al. (14) emphasize that older adults who have received higher education and possess a higher income are more predisposed to participating in a preventive health examination. These findings from Austria are consistent with results for the Czech Republic. In particular, our study underlines that the level of average wages, GDP and the unemployment rate affect the willingness of the 65+ population in the Czech Republic to undergo preventive check-ups.

Due to the lack of research focusing on the 65+ population, we will compare the results for the total population with other international scientific studies. Comparing our results for the total population with other scientific studies, they are similar to results published by Janssen et al. (20) and Al-Hanawi and Chirwa (23), that important factors that enhance the probability of undergoing a preventive health check-up comprise age, education level, possession of insurance, and being married. In a study published by Hoebel et al. (19), higher socioeconomic status, stronger social support, and increased use of outpatient care are highlighted. Similarly, Dryden et al. (18) found that low income, low socioeconomic status, unemployment, or low education are important indicators influencing preventive check-up motivation. In addition to these studies, Jørgensen et al. (21) underline socio-demographic and gender-specific factors. Labeit et al. (29) highlighted that socioeconomic variables can have varying impacts on the outcomes of different health check-ups. For instance, permanent household income in their study influenced only eyesight tests and dental screening.

Concerning employment/unemployment impact on preventive health examinations, the results of the scientific studies are inconclusive. While Vončina et al. (22) examine the whole population and find that the group of unemployed people attend fewer preventive check-ups than the group of employed people, Jørgensen et al. (21) reveals that employed people are 39% less likely to be frequent attendees compared to those who are unemployed. These studies focus on the general population. Our results for the population group 65+ are similar to the Jørgensen et al. (21) results for the whole population. The higher level of unemployment in the general population has a positive impact on the 65+ population in terms of attendance at GP health check-ups.

### 4.3 Strengths and weaknesses

Both strengths and weaknesses are mainly related to the data and methodology used. As far as the data is concerned, the strengths are the actual data obtained over a longer period of time for the whole Czech Republic, which tell us about the total number of preventive examinations performed on clients registered with the largest health insurance company in the Czech Republic. These are data that are not publicly available. As the data are available for each year in the given time period, it was possible to perform a regression analysis.

On the other hand, however, this data structure and type of analysis is also a weakness of the presented research, as it does not allow for the inclusion of variables that are not available for a given population on an annual basis in the period 2010–2019. There are also other factors that may influence the frequency of preventive examinations, such as occupation, health literacy, perceived health status, and others. However, these data are individual and their availability for the population analyzed and all insured citizens is not available. Our study works with macroeconomic data and data obtained from the largest insurance company in the country.

The existence of multicollinearity in our dataset is a feature arising from the nature of national demographic and macroeconomic data. These variables, such as income levels, education levels, employment status, and age distribution, are often interrelated because they reflect underlying social, economic, and cultural factors that tend to vary across regions, making it unrealistic to completely separate the effects of each variable. Eliminating multicollinearity by removing some variables reduces the representativeness of the model in terms of the theoretical model (with all explanatory variables advocated). Therefore, when interpreting the (partial) regression parameters of the three sub-models, we focused on the descriptive interpretation and the signs of the regression coefficients, rather than specific values. In this case, the values of the regression coefficients may be imprecise; it is more appropriate to focus on the signs of these coefficients in the complex theoretical model and in the sub-models. Specifically, whether, if the independent variable is increasing, we can observe a decrease or an increase in the dependent variable.

The presented analysis focuses on the Czech Republic as a whole, which represents also its weakness. According to some studies, there are significant differences between regions and it makes sense to make a regional comparison and analysis. For example, Al-Hanawi and Chirwa (23) underline that differences can be observed due to socioeconomic inequalities between regions and locations. Based on their research, the primary factors contributing to the associated inequality were income and education. As was mentioned in the Introduction, the current distribution of GP supply is relatively even in the Czech Republic. However, the provision of these services can be affected by the retirement of doctors from the system due to their age, particularly in rural areas (10). We can observe also other geographical health inequalities within the Czech Republic and its 14 regions. Life expectancy varies significantly within the 77 districts of the Czech Republic. The differences are likely to reflect a varying quality of services, the citizens' health condition, the level of health literacy, and the prevalence of risk factors and behaviors. Regarding regions in the Czech Republic, regional differences in the age structure were found of physicians and population at the level of regions, while when looking at regions by type of urban vs. rural, more differences were found in the age composition of the population (10).

### 4.4. Implication for research and practice

Analyzing the behavior of the 65+ population and the factors that influence it is important for several reasons, such as working life and retirement age, increasing life expectancy and a focus on healthy lifestyles for seniors. Health screenings can make a significant contribution to extending the healthy working lives of the 65+ generation.

Regarding the research implications, it would certainly make sense to focus on regional differences also with regard to regional socio-economic disparities. Subsequent research will therefore also focus on spatial analysis. At the same time, it makes sense to continue exploring motivations in the 65+ population in more depth. This generation will become increasingly important in view of the prolongation of active and healthy life, as well as the aging of the population.

Concerning the 65+ group, Patzelt et al. (31) underlined a need to consider gender-specific requirements to encourage the participation of older adults in preventive services. Age-specific traits appear to be of minor significance. It is crucial to pay attention to the individual health status and life circumstances of potential participants, as these are the prime factors influencing motivation.

Dealing with the implications for practice and policy makers, preventive health check-ups are of key importance to the health of the population for several important reasons: early diagnosis and treatment, disease prevention, improving quality of life, reduced health care costs, and patient education. Regular monitoring and health care can reduce the incidence of serious diseases and improve the quality of life of individuals (32–35).

The significance of preventive check-ups is also underscored in the Czech Republic, considering that general practitioners are financed for their execution through performance payments, provided in addition to the basic capitation payment (36). This performance payment reflects specifically performed services like preventive check-ups, thus contributing to the financial motivation of doctors to regularly conduct general preventive check-ups.

Similarly like in Croatia (22) or Saudi Arabia (13), to achieve greater equity in the distribution of preventive healthcare services, the Czech healthcare system should consider vulnerable groups, including those with limited education or pensioners. The government could develop intervention programmes and strategies aimed at increasing health check-up participation levels among those groups, thereby reducing health inequalities and raising awareness of the benefits of undergoing such examinations.

Policymakers should consider promoting preventive check-ups in light of the aging population and the sustainability of health system financing. Preventive check-ups are important for both the general population and the group of seniors. Based on the results of our study, economic incentives for prevention in the 65+ population should be considered.

For example, seniors over 65 currently have financial support for certain vaccinations (e.g., influenza or pneumococcal infections). Such economic instruments and incentives could be introduced more widely, and sufficiently promoted to raise awareness among seniors.

## 5 Conclusion

This paper aimed to identify socioeconomic predictors of preventive health check-ups by a general practitioner in the Czech Republic, with a focus on selected age groups. The original dataset based on annual data for the years 2010–2019 provided by the biggest health insurance company in the Czech Republic, General Health Insurance Company, was used. To achieve the objectives, the methods of correlation and regression analysis were used. Two models were constructed and tested: (1) preventive examinations model (PRE model) and (2) preventive examinations in age group 65+ model (PRE65 model).

The results indicate that socioeconomic factors, such as GDP, average wage, and unemployment, have a significant impact on the attendance of preventive check-ups. However, demographic and geographic factors, including population size, average age, and proportion of urban population, also play a significant role in models that focus on the total population. It is important to note that urban and rural disparities can affect access to healthcare resources. The educational level of the population, particularly those with a university degree, has a positive impact on preventive examinations.

The results of the analysis also showed that there is a difference between the general population group and the 65+ population group. Economic factors are much more important in the 65+ population group. Therefore, government interventions and health policies promoting prevention should consider using appropriate incentive policy instruments targeting this group to prolong active life in older age. The study only examined certain factors; however, there are numerous other factors that can impact the frequency of preventive health check-ups, such as occupation, health literacy, perceived health status, and others. Regarding the analyzed indicators, the study findings indicate that certain predictors are associated with vulnerable populations. Recommendations arising from our study can be directed to policy makers who should strengthen prevention activities, especially for this group.

## Data Availability

The raw data supporting the conclusions of this article will be made available by the authors, without undue reservation.
